# Pulmonary Bacterial Communities in Surgically Resected Noncystic Fibrosis Bronchiectasis Lungs Are Similar to Those in Cystic Fibrosis

**DOI:** 10.1155/2012/746358

**Published:** 2012-02-08

**Authors:** Heather Maughan, Kristopher S. Cunningham, Pauline W. Wang, Yu Zhang, Marcelo Cypel, Cecilia Chaparro, D. Elizabeth Tullis, Thomas K. Waddell, Shaf Keshavjee, Mingyao Liu, David S. Guttman, David M. Hwang

**Affiliations:** ^1^Centre for the Analysis of Genome Evolution & Function, University of Toronto, Toronto, ON, Canada M5S 3B2; ^2^Department of Cell & Systems Biology, University of Toronto, Toronto, ON, Canada M5S 3G5; ^3^Department of Laboratory Medicine & Pathobiology, University of Toronto, Toronto, ON, Canada M5S 1A8; ^4^Latner Thoracic Surgery Research Laboratories, University Health Network, Toronto, ON, Canada; ^5^Division of Respirology, St. Michael's Hospital and University of Toronto, Toronto, ON, Canada; ^6^Department of Pathology, Toronto General Hospital, University Health Network, Room 11E423, 200 Elizabeth Street, Toronto, ON, Canada M5G 2C4

## Abstract

*Background*. Recurrent bacterial infections play a key role in the pathogenesis of bronchiectasis, but conventional microbiologic methods may fail to identify pathogens in many cases. We characterized and compared the pulmonary bacterial communities of cystic fibrosis (CF) and non-CF bronchiectasis patients using a culture-independent molecular approach. *Methods*. Bacterial 16S rRNA gene libraries were constructed from lung tissue of 10 non-CF bronchiectasis and 21 CF patients, followed by DNA sequencing of isolates from each library. Community characteristics were analyzed and compared between the two groups. *Results*. A wide range of bacterial diversity was detected in both groups, with between 1 and 21 bacterial taxa found in each patient. *Pseudomonas* was the most common genus in both groups, comprising 49% of sequences detected and dominating numerically in 13 patients. Although *Pseudomonas* appeared to be dominant more often in CF patients than in non-CF patients, analysis of entire bacterial communities did not identify significant differences between these two groups. *Conclusions*. Our data indicate significant diversity in the pulmonary bacterial community of both CF and non-CF bronchiectasis patients and suggest that this community is similar in surgically resected lungs of CF and non-CF bronchiectasis patients.

## 1. Introduction

Bronchiectasis is a heterogenous condition characterized by chronic infection/inflammation and irreversible abnormal dilatation of the airways. Generally classified into cystic fibrosis (CF) and non-CF disease, bronchiectasis was associated with a high mortality rate in the preantibiotic era [1]. While the outcome of bronchiectasis, both CF and non-CF, has improved significantly, non-CF bronchiectasis (hereafter referred to simply as bronchiectasis) nevertheless remains a significant cause of respiratory morbidity that necessitates surgical resection and even lung transplantation in some patients.

While the cause of CF has been pinpointed to mutations in a single gene, non-CF bronchiectasis may be associated with a wide range of different conditions including ciliary dysmotility syndromes, immune deficiencies, and chronic obstructive pulmonary disease (COPD) and, in many cases, is idiopathic [2]. The common underlying pathogenic mechanism, however, appears to be recurrent airway infection and inflammation resulting in scarring and permanent dilatation of the airways.

Given the centrality of airway infection in the pathogenesis of bronchiectasis, a number of studies have endeavored to identify bacterial pathogens present in these patients, using conventional microbiologic methods [3–11]. Such studies have typically found the most common pathogens to be nontypable *Haemophilus influenzae* and *Pseudomonas aeruginosa*, followed by a handful of others including *Moraxella catarrhalis*, *Streptococcus pneumoniae*, *Staphylococcus aureus*, and coliforms. Nontuberculous mycobacteria have also been reported with variable frequency. Such studies suggest that, despite seemingly similar pathophysiologic mechanisms, there may be differences in the bacterial species infecting CF and non-CF bronchiectasis patients. For example, while *H. influenzae* is the most common pathogen in adult non-CF bronchiectasis (approximately 40% of cases), it is far less prevalent in adult CF patients [1, 12]. On the other hand, *P. aeruginosa* is far more commonly isolated among adult CF patients than from bronchiectasis patients (approximately 80% versus 18%, resp.) [1, 12]. Further, the *Burkholderia cepacia* complex, a group of organisms associated with significantly impaired outcome in CF patients, infects bronchiectasis patients quite infrequently in comparison to CF patients [13]. These observations suggest that despite similar changes in the disease morphology within the airways, other factors and mechanisms may be at play in determining what organisms are able to infect each group of patients.

These studies, however, almost certainly underestimate the bacterial diversity of the lungs in patients with bronchiectasis, given that 30–40% of sputum samples may fail to grow pathogenic bacteria in conventional culture, despite being of good quality and purulent [2]. Such studies, therefore, may underestimate the extent of diversity and variation within the pulmonary bacterial flora associated with bronchiectasis. Application of the methods and strategies of an emerging field of study known as metagenomics may help address these issues. Metagenomics is the study of communities of organisms using genetic material taken directly from their natural environment [14]. Metagenomics' power comes from its ability to assess community structure and dynamics without needing first to isolate and propagate organisms in the laboratory. Free of the constraints and limitations imposed by culture-based methods, it provides a semiquantitative assessment of community diversity. Communities can be studied without any prior knowledge of their composition and without the need for specific selective conditions, by isolating and characterizing DNA directly from the metagenome—that is, the combined genomes of all species present in the specimen of interest—bypassing the need for laboratory isolation and cultivation. The potential power of metagenomics to investigate questions related to health and disease has been increasingly recognized in recent years, as evidenced, for example, by the recent launches of the International Human Microbiome Consortium (http://www.human-microbiome.org/), the Human Microbiome Project (HMP), and other similar initiatives around the world [15, 16].

A growing number of studies using culture-independent molecular strategies for detection of bacterial species have demonstrated striking diversity of the pulmonary bacterial community in CF patients [17–27]. However, few if any similar studies have been done to characterize pulmonary bacterial communities in bronchiectasis. We, therefore, endeavored to profile the diversity of the pulmonary bacterial community in patients with bronchiectasis by sequencing bacterial 16S rRNA gene libraries constructed using surgically excised lung tissue from 10 patients with bronchiectasis; we compared these communities to those seen in 21 patients with CF.

## 2. Methods

### 2.1. Patients and Sample Collection

Protocols for specimen collection, storage, and use were approved by the University Health Network (UHN) Research Ethics Board. Under aseptic conditions, tissue specimens were collected from patients with an established diagnosis of either CF or non-CF bronchiectasis undergoing surgical resection (pneumonectomy) or lung transplantation at the Toronto General Hospital between 1998 and 2007. Peripheral lung tissues were collected under sterile conditions intraoperatively, flash frozen in liquid nitrogen, and stored at −80°C. Specimens from 10 patients with non-CF bronchiectasis and 21 randomly selected CF patients were analyzed (Table 1). All specimen processing was performed blinded to patient data. Clinical data were extracted from electronic patient records at UHN.

### 2.2. DNA Extraction and 16S rRNA Gene Amplification and Sequencing

Genomic DNA was extracted using the Direct PCR DNA Extraction System (Viagen Biotech, Los Angeles, CA). PCR reactions and negative controls were prepared in a separate room from where amplifications were conducted. Amplification of 16S rRNA was performed using the 8F (5′-AGAGTTTGATCCTGGCTCAG) and 806R (5′-GGACTACCAGGGTATCTAAT) PCR primers, as follows: 1 cycle (95°C, 5 min); 30 cycles (95°C, 30 sec; 55°C, 30 sec; 72°C, 30 sec); 1 cycle (72°C, 8 min). Amplicon libraries for each specimen were constructed using the TA cloning kit (Invitrogen, Carlsbad, CA) and plated on LB media. Plasmids from individual isolates were purified using the Pure Link Quick Plasmid Miniprep kit (Invitrogen). DNA sequencing was performed on an Applied Biosystems 3130XL sequencer (Analytical Genetics Technology Centre, Toronto) on up to 48 isolates initially, followed by preliminary assessment of diversity using BLAST [28]. Specimens containing more than one taxon were subjected to additional sequencing. Chimeras were identified by BLAST analysis of end sequences and excluded from analysis.

### 2.3. Data Analysis

Sequences were analyzed using the Qiime community analysis pipeline [29]. Sequences were clustered into operational taxonomic units (OTUs) using a 97% identity threshold. One sequence representing the most abundant from each OTU was chosen for taxonomic classification using the RDP classifier [30]. Taxonomy classifications with a support level greater than or equal to 0.7 were retained; this resulted in not all sequences being classified to the most resolved (i.e., genus) level. All whole bacterial community comparisons included all sequences regardless of the level to which their taxonomy was assigned; however, for the identification and analysis of numerically dominant taxa, we chose only those OTUs classified to the genus level since this has the most functional information. OTU taxonomy classifications were used to construct the OTU table used in rarefaction analysis, heatmap construction, and distance calculations. Rarefaction analysis was performed in Qiime [29], using 100 replicate samplings of the sequences for subsequent estimation of alpha diversity. Calculation of UniFrac metrics [31] and principal components analyses were done in Qiime [29]. Clustering for heatmap presentation was performed in R [32].

## 3. Results

### 3.1. Bacterial Community Diversity of the Lungs in Patients with Bronchiectasis and CF

In total, 955 16S rRNA gene sequences were obtained from lung tissue specimens of 10 patients with bronchiectasis (mean 96 ± 29  (sd) per patient) and 1387 16S rRNA gene sequences from lung tissue specimens of 21 patients with CF (mean  66 ± 24  (sd) sequences per patient). Etiologies underlying the diagnoses of bronchiectasis included postinfectious (4 patients), immune dysfunction (3 patients), chronic obstructive pulmonary disease (1 patient), and idiopathic (2 patients). Taxonomic classification identified 37 unique operational taxonomic units (OTUs) representing 20 genera in bronchiectasis patients and 51 unique OTUs representing 29 genera in CF patients, with 16.7 ± 2.9 OTUs representing 9.1 ± 1.7 genera per patient in bronchiectasis and 11.5 ± 6.0 OTUs representing 6.8 ± 3.5 genera per patient in CF (range 1–21) (Table 1). These OTUs represented 4 phyla, Proteobacteria being most numerous in both groups (Table 2), and included many taxa not typically reported as constituents of the respiratory tract microflora (Table 3).

*Pseudomonas* was the most prevalent genus overall, with a total of 9 OTUs being detected in 30 of 31 patients, including all 10 bronchiectasis patients, and accounting for 49% of all sequences (Table 3). However, *Pseudomonas* was the numerically predominant genus (i.e., the genus represented by the largest number of 16S rRNA sequences) in only 13 patients, with various other taxa predominating in the other 18 patients. *Pseudomonas *was numerically predominant more frequently in CF patients (11/21 patients) than in bronchiectasis (2/10 patients), although this difference was not statistically significant (*P* = 0.18, 2-sided Fisher's Exact Test). Sequences from other genera known to colonize adult CF patients were also detected, including *Stenotrophomonas* and *Staphylococcus* in both patient groups. *Haemophilus* was detected in only 2 patients with CF and 1 patient with bronchiectasis. Interestingly, *Burkholderia* sequences were detected at low levels in 4 patients with bronchiectasis.

**Figure 1:**
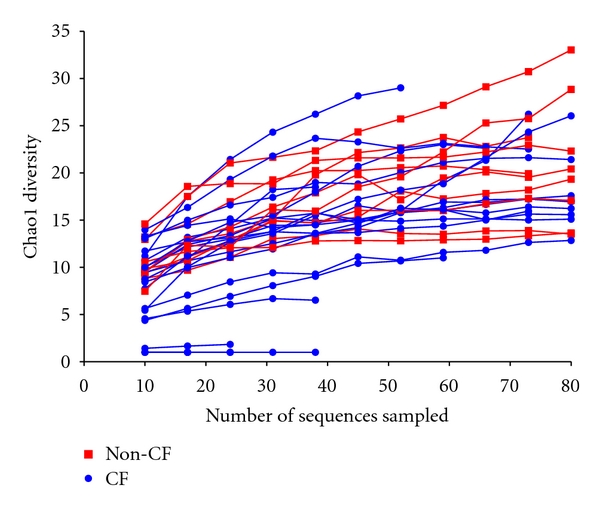
Rarefaction curves estimating the effect of sampling on Chao1 diversity measurements. The sequence data were resampled 100 times in Qiime [29], and, for each sample, the Chao1 diversity measurement was estimated. Red lines indicate bronchiectasis patients, whereas blue lines indicate cystic fibrosis (CF) patients.

We used rarefaction analysis to determine whether the number of sequences we obtained from each patient was a sufficient sample of the sequence diversity present. Rarefaction analysis works by repeated sampling of the data; with each of these samples, the OTU diversity is calculated and plotted to determine whether the observed diversity increases with more sampling. If the sampling saturates the diversity present, then the curve will plateau, whereas if there is additional diversity yet to be discovered, the curve will continue to rise. Considering the relatively low number of sequences we sampled for each patient, most of the rarefaction curves indicate sufficient sampling of the diversity present (Figure 1). Although there are a few patient curves that are undersampled (i.e., that continue to increase), these are not restricted to bronchiectasis or CF patients alone, indicating that any potential differences in bronchiectasis versus CF community structure are unlikely to be due to insufficient sampling.

### 3.2. Comparison of Pulmonary Bacterial Communities in Bronchiectasis and CF

**Figure 2:**
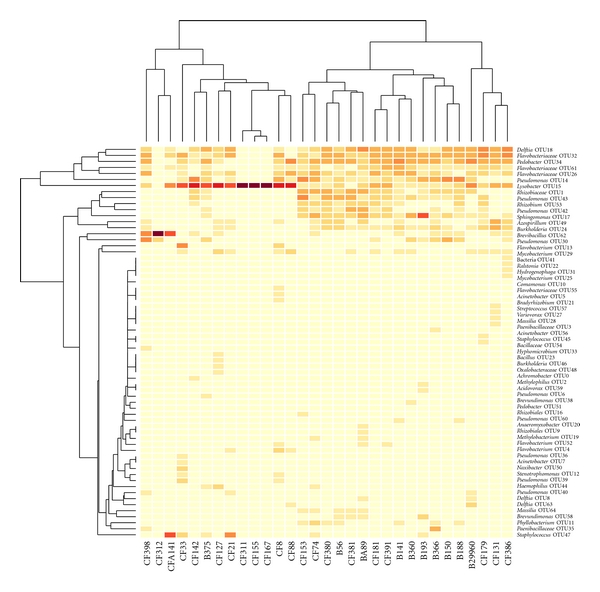
Heatmap showing clustering of bacterial communities and similarities in operational taxonomic unit (OTU) distribution among patients. Sample numbers are listed horizontally along the bottom; “B” indicates bronchiectasis samples, and “CF” indicates CF samples. OTUs and their taxonomic classifications are listed vertically on the right. Darker colors indicate higher abundance; lighter colors indicate low abundance or absence of a particular OTU.

We compared the bacterial communities between bronchiectasis and CF patients in three ways: clustering of community profiles, clustering of community distances, and principal components analysis. Community profiles were clustered and are represented in the heatmap shown in Figure 2. Although there is a small cluster of bronchiectasis-derived communities (B188, B150, B366, and B193), those not clustered together are interspersed with the CF communities.

**Figure 3:**
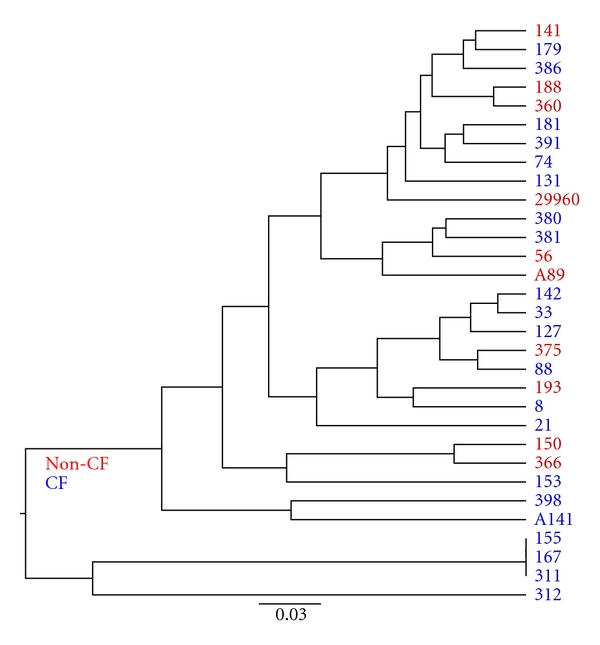
Unweighted pair group method with arithmetic mean (UPGMA) tree inferred using UniFrac distances. Red and blue names indicate bronchiectasis and CF, respectively.

 The heatmap clustering is informative for comparing patients based on their community profiles but does not consider sequence differences within or between OTUs. Therefore, we also compared the sequence divergence in these community profiles within a phylogenetic framework by calculating the normalized weighted UniFrac distance [31] and using these distances to infer a UPGMA phylogenetic tree (Figure 3). As is clear from the grouping of different patients in the tree, there exist no clear differences between bacterial communities from bronchiectasis and CF patients. In fact, when sequence divergence is taken into account, the small cluster of bronchiectasis patients noted in Figure 2 is no longer present (Figure 3).

**Figure 4:**
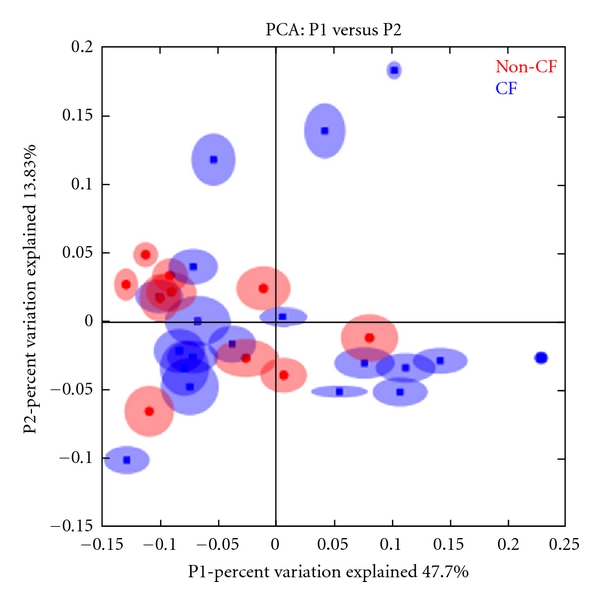
Principal components analysis (PCA) of bronchiectasis (red) and CF patients (blue).

Finally, we used principal components analysis (PCA) to identify whether differences exist between bronchiectasis and CF communities. PCA is useful for removing correlations in the data that may obscure the detection of differences that exist due to a diagnosis of bronchiectasis versus CF (e.g., age of patient). The results from PCA agree with the results from the other two methods described above, namely, that significant differences in bacterial community structure could not be found when comparing bronchiectasis to CF patients (Figure 4).

## 4. Discussion

Bacterial infection is a major cause of morbidity in individuals with CF and non-CF bronchiectasis. Management of bacterial infections historically has been based primarily on culture-based isolation of a limited number of species considered pathogenic, but there is growing appreciation of the polymicrobial nature of pulmonary infections particularly in the setting of chronic illnesses such as CF, and increasing evidence indicates that interactions between species may alter virulence properties or antibiotic susceptibility of individual species [33]. Better understanding of such interactions and their potential clinical impacts requires, as a first step, more complete characterization of the composition of the microbial community of the lungs. We describe here the application of culture-independent metagenomic-based strategies to the study of the bacterial communities of the lung. While a number of studies have applied similar strategies to the study of respiratory microbiota in CF patients [17–27], this study is, to our knowledge, the first reporting culture-independent sequence-based profiling of bacterial species directly from lung tissue of patients with non-CF bronchiectasis. Our data demonstrate significant diversity in the pulmonary bacterial communities of both CF and bronchiectasis patients but did not detect significant differences in overall community composition between these two groups of patients.

Proteobacteria predominated in both CF and non-CF groups (Tables 2 and 3), with *Pseudomonas* being the most prevalent genus overall. *Pseudomonas* was numerically predominant in 2 of 10 patients with bronchiectasis, which appears to be in keeping with previously reported rates of isolation of *P. aeruginosa* from bronchiectasis patients in the neighborhood of 18% [4]. Interestingly, however, while *Pseudomonas* was numerically predominant in 2 bronchiectasis patients, it was identified as a minor constituent of the bacterial community in the remaining 8 bronchiectasis patients. On the other hand, whereas *H. influenzae* is typically cultured from approximately 40% of bronchiectasis patients, it was detected in only 1 of 10 bronchiectasis patients in this study. The reasons for these discrepancies between our culture-independent data and previous culture-based studies may be several-fold. Given that the present study examined tissue samples from resected lung specimens including many from patients undergoing lung transplantation, the bacterial community profiles generated here most likely represent those present in end-stage lung disease, and, together with the relatively small number of specimens profiled here, therefore may not be completely representative of the microflora in earlier stages of disease process. Further, this study examined lung tissue samples, whereas previous studies of bacterial species in bronchiectasis were based largely on cultures of sputum samples. Because *H. influenzae* frequently colonizes the upper respiratory tract, it may be the case that previous sputum-based studies have overestimated the prevalence of *H. influenzae* in the lung itself. Finally, the high frequency of *Pseudomonas* detection in our bronchiectasis data set compared to culture-based studies could possibly be due to inhibition of growth of *Pseudomonas* in culture by interspecies interactions with other, more prevalent species present within sputum samples. Such inhibition could possibly account for at least part of the large proportion of sputum samples from bronchiectasis patients in which no pathogens are detected by conventional culture methods [2]. Similar factors could also account for our detection of *Burkholderia* in 4 of 10 bronchiectasis patients, whereas a previous culture-based study failed to isolate any Burkholderia from sputum samples of patients with bronchiectasis [13].

Comparison of pulmonary bacterial community profiles from bronchiectasis patients with those obtained from CF patients using three different methods found no significant differences in community structure between the two groups. At this point, it is uncertain whether this is simply a function of the fact that the specimens analyzed in this study were derived from end-stage lungs (both CF and non-CF) and whether the pulmonary bacterial communities of CF and bronchiectasis patients at earlier stages may demonstrate more significant differences. Longitudinal studies profiling respiratory samples from bronchiectasis patients in comparison with CF patients over time are needed to shed light on this question.

In summary, our data indicate significant diversity in the bacterial community of the lungs in both CF and non-CF bronchiectasis patients but did not detect significant differences in the bacterial community structure between these two groups. The application of culture-independent methods, such as those described here, in concert with conventional culture-based studies should help significantly advance understanding of the microbiology of the bronchiectatic airway.

## Figures and Tables

**Table 1:** Sequences and OTUs for each patient.

Patient	Source*	Number of rRNA sequences	Number of OTUs (number of *Pseudomonas* OTUs)	Number of genera
A141	CF	39	6 (1)	5
A89	B	81	21 (3)	12
8	CF	54	17 (3)^+^	8
21	CF	38	11 (2)^+^	6
33	CF	81	14 (4)^+^	8
56	B	101	21 (3)	11
74	CF	79	20 (3)	13
88	CF	59	8 (1)^+^	4
127	CF	86	14 (1)^+^	8
131	CF	89	14 (1)^+^	11
141	B	98	17 (4)	9
142	CF	87	10 (2)^+^	5
150	B	90	13 (2)	7
153	CF	81	15 (3)	7
155	CF	28	1 (1)^+^	1
167	CF	43	1 (1)^+^	1
179	CF	63	14 (3)	9
181	CF	85	15 (3)^+^	8
188	B	86	15 (4)	8
193	B	77	16 (2)^+^	10
311	CF	18	1 (1)^+^	1
312	CF	27	2 (0)	2
360	B	86	13 (2)	7
366	B	77	18 (2)	10
375	B	84	15 (3)^+^	8
380	CF	91	15 (3)	8
381	CF	94	19 (3)	11
386	CF	70	16 (2)	11
391	CF	79	14 (3)	7
398	CF	76	13 (2)	8
29960	B	175	18 (4)	9

*CF denotes cystic fibrosis patients; B denotes bronchiectasis.

rRNA: ribosomal ribonucleic acid; OTU: operational taxonomic unit.

^+^ *Pseudomonas* is the numerically predominant taxon.

**Table 2:** Phyla detected in lungs.

Phylum	% sequences in CF patients	% sequences in bronchiectasis patients
Proteobacteria	57.3	64.6
Bacteroidetes	37.6	30.2
Actinobacteria	3.5	0.1
Firmicutes	1.6	5.1

**Table 3:** Taxonomic classification of all sequences.

Taxonomy	Level of resolution	Phylum	Total sequences
*Pseudomonas* OTU14	Genus	Proteobacteria	490
*Delftia* OTU18	Genus	Proteobacteria	260
*Flavobacteriaceae* OTU32	Family	Bacteroidetes	227
*Delftia* OTU37	Genus	Proteobacteria	207
*Flavobacteriaceae* OTU26	Family	Bacteroidetes	148
*Flavobacteriaceae* OTU61	Family	Bacteroidetes	118
*Pseudomonas* OTU42	Genus	Proteobacteria	110
*Rhizobiaceae* OTU1	Family	Proteobacteria	102
*Lysobacter* OTU15	Genus	Proteobacteria	98
*Burkholderia* OTU24	Genus	Proteobacteria	72
*Pedobacter* OTU34	Genus	Bacteroidetes	70
*Brevibacillus* OTU62	Genus	Firmicutes	66
*Azospirillum* OTU49	Genus	Proteobacteria	63
*Rhizobium* OTU53	Genus	Proteobacteria	45
*Pseudomonas* OTU43	Genus	Proteobacteria	42
*Sphingomonas* OTU17	Genus	Proteobacteria	36
*Staphylococcus* OTU47	Genus	Firmicutes	25
*Pseudomonas* OTU30	Genus	Proteobacteria	23
*Flavobacterium* OTU13	Genus	Bacteroidetes	20
*Phyllobacterium* OTU11	Genus	Proteobacteria	14
*Brevundimonas* OTU58	Genus	Proteobacteria	10
*Massilia* OTU64	Genus	Proteobacteria	10
*Paenibacillaceae* OTU35	Family	Firmicutes	9
*Delftia* OTU63	Genus	Proteobacteria	7
*Haemophilus* OTU44	Genus	Proteobacteria	6
*Pseudomonas* OTU39	Genus	Proteobacteria	6
*Delftia* OTU8	Genus	Proteobacteria	4
*Flavobacterium* OTU4	Genus	Bacteroidetes	4
*Pseudomonas* OTU40	Genus	Proteobacteria	4
*Flavobacterium* OTU52	Genus	Bacteroidetes	3
*Naxibacter* OTU50	Genus	Proteobacteria	3
*Stenotrophomonas* OTU12	Genus	Proteobacteria	3
*Brevundimonas* OTU38	Genus	Proteobacteria	2
*Methylobacterium* OTU19	Genus	Proteobacteria	2
*Pseudomonas* OTU60	Genus	Proteobacteria	2
*Rhizobiales* OTU16	Order	Proteobacteria	2
*Achromobacter* OTU0	Genus	Proteobacteria	1
*Acidovorax* OTU59	Genus	Proteobacteria	1
*Acinetobacter* OTU5	Genus	Proteobacteria	1
*Acinetobacter* OTU56	Genus	Proteobacteria	1
*Acinetobacter* OTU7	Genus	Proteobacteria	1
*Anaeromyxobacter* OTU20	Genus	Proteobacteria	1
*Bacillaceae* OTU54	Family	Firmicutes	1
*Bacillus* OTU23	Genus	Firmicutes	1
Bacteria OTU41	Domain	Bacteria	1
*Bradyrhizobium* OTU21	Genus	Proteobacteria	1
*Burkholderia* OTU46	Genus	Proteobacteria	1
*Comamonas* OTU10	Genus	Proteobacteria	1
*Flavobacteriaceae* OTU55	Family	Bacteroidetes	1
*Hydrogenophaga* OTU31	Genus	Proteobacteria	1
*Hyphomicrobium* OTU33	Genus	Proteobacteria	1
*Massilia* OTU28	Genus	Proteobacteria	1
*Methylophilus* OTU2	Genus	Proteobacteria	1
*Mycobacterium* OTU25	Genus	Actinobacteria	1
*Mycobacterium* OTU29	Genus	Actinobacteria	1
*Oxalobacteraceae* OTU48	Family	Proteobacteria	1
*Paenibacillaceae* OTU3	Family	Firmicutes	1
*Pedobacter* OTU51	Genus	Bacteroidetes	1
*Pseudomonas* OTU36	Genus	Proteobacteria	1
*Pseudomonas* OTU6	Genus	Proteobacteria	1
*Ralstonia* OTU22	Genus	Proteobacteria	1
*Rhizobiales* OTU9	Order	Proteobacteria	1
*Staphylococcus* OTU45	Genus	Firmicutes	1
*Streptococcus* OTU57	Genus	Firmicutes	1
*Variovorax* OTU27	Genus	Proteobacteria	1

OUT: operational taxonomic unit.
